# Supplementary material for: Modelling Skylarks (Alauda arvensis) to Predict Impacts of Changes in Land Management and Policy: Development and Testing of an Agent-Based Model
Source: PLoS One. 2013 Jun 6;8(6):e65803. doi: 10.1371/journal.pone.0065803 (PMC3675089; doi:10.1371/journal.pone.0065803)
Supplement: Supporting Information S4 — The skylark ODdox as a zipped archive. (ZIP) [file pone.0065803.s004.zip › Skylark_ODdox/hedgebanks_8cpp.html]

ALMaSS Skylark ODdox: hedgebanks.cpp File Reference


ALMaSS Skylark ODdox
 2.0


- Main Page
- Related Pages
- Classes
- Files

- File List
- File Members

Macros |
Variables

hedgebanks.cpp File Reference

`#include "ls.h"`  
`#include "map_cfg.h"`  
`#include <math.h>`

|  |  |
| --- | --- |
| Macros | |
| #define | \_CRT\_SECURE\_NO\_DEPRECATE |
| #define | HB\_MAGIC   1000 |
| #define | HB\_MAGIC\_COLOR   999 |
| #define | HB\_MAGIC\_PAINTER\_BIT   0x40000000 |

|  |  |
| --- | --- |
| Variables | |
| static CfgFloat | l\_map\_art\_hb\_core\_thres ("MAP\_ART\_HB\_STRIPE\_THRES", CFG\_CUSTOM, 0.4) |
| static CfgInt | l\_map\_art\_hb\_nums ("MAP\_ART\_HB\_NUMS", CFG\_CUSTOM, 3) |
| static CfgFloat | l\_map\_art\_hb\_seg\_len ("MAP\_ART\_HB\_SEG\_LEN", CFG\_CUSTOM, 100.0) |
| static CfgInt | l\_map\_art\_hb\_tran\_prob ("MAP\_ART\_HB\_TRAN\_PROB", CFG\_CUSTOM, 30) |
| static CfgFloat | l\_map\_art\_hb\_width ("MAP\_ART\_HB\_WIDTH", CFG\_CUSTOM, 0.3) |

---

## Macro Definition Documentation

#define \_CRT\_SECURE\_NO\_DEPRECATE

#define HB\_MAGIC   1000

Referenced by Landscape::hb\_Add(), Landscape::hb\_AddNewHedgebanks(), Landscape::hb\_DownPolyNumbers(), Landscape::hb\_HasOtherNeighbour(), Landscape::hb\_MarkTheBresenhamWay(), and Landscape::hb\_UpPolyNumbers().

#define HB\_MAGIC\_COLOR   999

Referenced by Landscape::hb\_ClearPolygon(), Landscape::hb\_PaintBorder(), and Landscape::hb\_PaintWhoHasNeighbourColor().

#define HB\_MAGIC\_PAINTER\_BIT   0x40000000

Referenced by Landscape::hb\_MarkTopFromLocalMax(), Landscape::hb\_MaxUnpaintedNegNeighbour(), and Landscape::hb\_ResetColorBits().

---

## Variable Documentation

CfgFloat l\_map\_art\_hb\_core\_thres("MAP\_ART\_HB\_STRIPE\_THRES", CFG\_CUSTOM, 0.4)

static

Referenced by Landscape::hb\_Add().

CfgInt l\_map\_art\_hb\_nums("MAP\_ART\_HB\_NUMS", CFG\_CUSTOM, 3)

static

Referenced by Landscape::hb\_GenerateHBPolys(), and Landscape::hb\_StripingDist().

CfgFloat l\_map\_art\_hb\_seg\_len("MAP\_ART\_HB\_SEG\_LEN", CFG\_CUSTOM, 100.0)

static

Referenced by Landscape::hb\_MarkTheBresenhamWay().

CfgInt l\_map\_art\_hb\_tran\_prob("MAP\_ART\_HB\_TRAN\_PROB", CFG\_CUSTOM, 30)

static

Referenced by Landscape::hb\_StripingDist().

CfgFloat l\_map\_art\_hb\_width("MAP\_ART\_HB\_WIDTH", CFG\_CUSTOM, 0.3)

static

Referenced by Landscape::hb\_Add(), and Landscape::hb\_MarkTheBresenhamWay().


- CJT
- MSVC
- ALMaSS Working Source
- Landscape
- hedgebanks.cpp
- Generated on Thu Jan 10 2013 13:15:35 for ALMaSS Skylark ODdox by
   1.8.1.1
